# Invisible CMOS Camera Dazzling for Conducting Adversarial Attacks on Deep Neural Networks

**DOI:** 10.3390/s25072301

**Published:** 2025-04-04

**Authors:** Zvi Stein, Adir Hazan, Adrian Stern

**Affiliations:** School of Electrical and Computer Engineering, Ben-Gurion University of the Negev, Beer-Sheva 8410501, Israel; tzviste@post.bgu.ac.il (Z.S.); stern@bgu.ac.il (A.S.)

**Keywords:** adversarial attack, PSF, rolling shutter, CMOS

## Abstract

Despite the outstanding performance of deep neural networks, they remain vulnerable to adversarial attacks. While digital domain adversarial attacks are well-documented, most physical-world attacks are typically visible to the human eye. Here, we present a novel invisible optical-based physical adversarial attack via dazzling a CMOS camera. This attack involves using a designed light pulse sequence spatially transformed within the acquired image due to the camera’s shutter mechanism. We provide a detailed analysis of the photopic conditions required to keep the attacking light source invisible to human observers while effectively disrupting the image, thereby deceiving the DNN. The results indicate that the light source duty cycle controls the tradeoff between the attack’s success rate and the degree of concealment needed.

## 1. Introduction

Deep Neural Networks (DNNs) have revolutionized the field of image analysis and processing, delivering state-of-the-art performance across a range of applications. However, these systems are inherently vulnerable to adversarial attacks [1], which introduce subtle perturbations to the input signal that cause the DNNs to make incorrect predictions. The concept of adversarial examples, commonly known as *attacked images*, was first introduced a decade ago by Szegedy et al. [2], demonstrating that DNNs could be easily misled by seemingly minor modifications to input images. Since then, numerous approaches for generating adversarial examples have been explored [3], highlighting the significant security concerns surrounding DNN-based systems.

The underlying mechanism for adversarial susceptibility lies in the way DNNs process images. Rather than learning the actual semantic content of the image, these networks often rely on superficial or spurious features for classification, as described by Goodfellow et al. as a “Potemkin village” of features [4]. This explains why two images that are visually indistinguishable from human vision can be classified differently by a DNN, revealing a vulnerability that adversarial attacks exploit. These attacks often aim to minimize the perturbations applied to an image so that the changes are not noticeable to the human eye while still causing a misclassification.

Adversarial attacks on DNNs can be divided into digital and physical attacks. While digital attacks manipulate image pixels, they often struggle to transfer to the physical world due to dynamic conditions and deployment challenges. Physical attacks alter real-world objects’ visual characteristics and pose a threat but are typically invasive, requiring visible changes that can be easily dismissed and detected by human vision. However, optical-based physical adversarial attacks are non-invasive and generate perturbations that mimic natural effects, making them harder to detect and better suited for real-world applications [5]. Despite advancements in imperceptibility, many of these attacks still have an obvious trace in the physical domain, limiting their effectiveness and feasibility, with achieving complete invisibility to the human eye remaining an unresolved challenge.

This paper introduces and demonstrates a novel optical-based physical adversarial attack that leverages the rolling shutter mechanism of CMOS sensors. The proposed attack is designed to be invisible in the physical domain, ensuring that the attacking light source remains undetectable to the scene observer. This involves a designed light pulse sequence spatially transformed during the image acquisition, effectively disrupting the camera’s image processing to deceive DNNs with a high attack success rate. Furthermore, our approach does not require precise alignment of the adversarial spatial pattern with the target object location, offering greater flexibility in real-world scenarios. A successful invisible attack is achieved when the beam of Attacking MOdulated Light Source (AMOLS) covers the camera aperture, such that the following are achieved:

1. The peak irradiance is sufficient to dazzle the sensor temporarily;
2. The average irradiance remains below the sensitivity threshold of the human eye.

The following summarizes the primary contributions of this work:

- We propose a physical domain adversarial attack on DNNs that receive images from a CMOS camera. The attack involves directing a light source toward the camera; however, the presence of the projected light is completely unnoticed by observers in the scene.
- We introduce an optical attack that is based on dazzling a camera sensor by sending short pulses. We investigate the effect of the projected pulses on the image captured by the CMOS camera. We evaluate the irradiance required to attack the image.
- We explore the relationship between the human eye’s ability to distinguish the attacking light source directed at the camera and the disruption of DNN performance caused by the influence of the pulsed laser beam. We analyze the photopic conditions required to ensure that the attacking light source remains invisible to human observers while still effectively disrupting the acquired image to mislead the classifier model.
- We evaluate the trade-off between the success of DNN attacks caused by dazzling pulses and their invisibility to the human eye. Our findings indicate that the duty cycle of the light source can be adjusted to manage the balance between the attack’s success rate and the level of concealment required.
- We present simulated and real experimental results to demonstrate the effectiveness of our attack.

## 2. Related Works

While most studies on adversarial attacks have focused on the digital domain, where perturbations are added to pixel values, growing efforts have expanded into the physical domain [6]. Examples of physical-world attacks typically include using adversarial objects or imaging system manipulations to fool DNN models. These modifications may include simple changes, such as adding elements like stickers, eyeglasses, earrings, and others to a real-world object [7], to more complex approaches. The more complex methods typically involve optical-based techniques [5], including temporarily projecting specifically crafted adversarial perturbations onto target objects [8], among others, or strategically illuminating target objects using infrared light sources [9]. Furthermore, synthesizing Three-Dimensional (3D) adversarial objects has been proposed to confuse classifier models [10], and imaging projection transformation in a 3D physical environment was demonstrated to deceive object detection systems effectively [11]. These examples highlight the growing applicability of adversarial attacks in real-world settings.

Recent studies on physical adversarial examples have increasingly focused on manipulating imaging systems themselves. For instance, Liu et al. [12] induced perturbations in the captured image through an electromagnetic injection attack. They focused on CCD sensors but noted that CMOS sensors, which have an independent measurement unit for each pixel, provide greater resilience to electromagnetic interference, making them more robust against such threats. Additionally, Duan et al. [13] employed a laser beam attack to create spatially tailored perturbations; however, they noted that this approach has a limited success rate in dynamic conditions. Many physical adversarial attacks require precise alignment of the adversarial spatial pattern with the target object placement. Moreover, Liu et al. [14] inject their attack after image acquisition, targeting the data lane between the camera sensor and the endpoint device. This requires physical access to the sensor-enabled system, which is practically infeasible in certain situations.

In this work, we develop an invisible camera dazzling attack that leverages the rolling shutter mechanism inherent in CMOS sensors. Unlike the continuous-wave operation of light sources, where the degree of dazzle on CMOS sensors can be depicted by the dazzling area or the number of saturated pixels [15], temporally modulated light can produce adjustable stripes in a captured image—introducing a unique approach to injecting adversarial spatial patterns. The rolling shutter effect is primarily studied in the context of mitigating distortions caused by fast-moving objects that approach the camera’s scanning frequency [16]. Accordingly, models have been developed to correct these distortions. Moreover, it was proposed that a smartphone camera can be used for visible light communications to detect and convert a temporal signal into spatial patterns by exploiting the rolling shutter effect of CMOS sensors [17].

**Figure 1:**
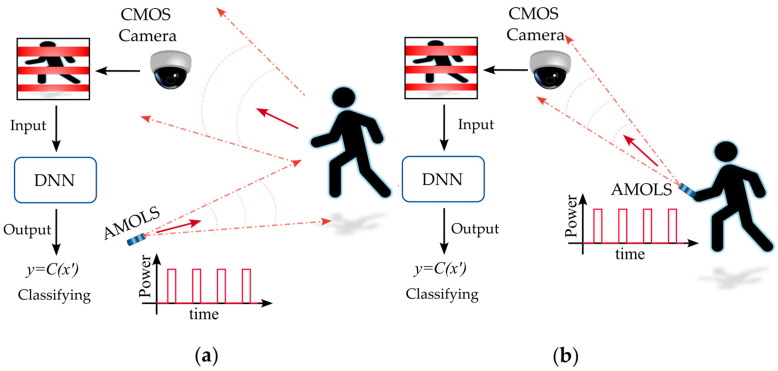
Practical physical-world adversarial attack. The attack can be carried out either (**a**) by temporally modulating a light source to illuminate the entire scene, which reflects light pulses onto the CMOS sensor, or (**b**) by directing a pulsed laser beam specifically at a CMOS sensor. The red arrows indicate the propagation direction of the light.

Adversarial attacks leveraging the rolling shutter mechanism have been introduced in references [18,19,20,21,22], where temporally modulated LEDs are used to illuminate a target object, as shown in Figure 1a. This results in distortions in the acquired image due to the camera’s row-wise scanning process. The first configuration [18] was introduced as a black-box backdoor attack on face recognition systems, where illuminating the entire scene induces perturbations employing the rolling shutter effect. While the first two studies [18,19] utilize programmable RGB LEDs, resulting in an adversarial signal with three adjustable components of Red, Green, and Blue, later work [20] demonstrated the use of a common commercial LED with a modulator to control the frequency of the emitted white light. In addition, further schemes [21,22] expanded the application of the white light attack method, showcasing the generalization and transferability of adversarial samples across different models and tasks, including traffic sign recognition systems and lane detection models. However, these approaches require comprehensive illumination of the whole scene and usually fail to remain invisible to the human eye. Despite the light pulse sequence being designed with a modulation frequency that prevents flickering perceived by the human eye, the illumination source still appears steady and is not stealthy to the human observer in the scene.

Here, we propose to employ an AMOLS beam that directly illuminates the camera’s aperture as shown in Figure 1b, taking advantage of the rolling shutter’s scanning process to induce real-world adversarial perturbations on the acquired image. Since the pulsed light beam is directed toward the camera rather than reflecting off a target object (see Figure 1), the average power requirements are significantly reduced compared to previous methods. While Kohler et al. [23] and Yan et al. [24] introduced such *a camera* attack utilizing a laser and exploiting the rolling shutter mechanism, their approaches still leave an obvious trace of the attack in the physical domain and remain visible to the human eye.

Since the integration time of the human eye is significantly longer than the acquisition time of each row in a rolling shutter scanning process, a high-frequency modulated signal is seen as continuous by the human eye. If denoting the duty cycle of the AMOLS as D, the intensity perceived by the human eye can be expressed as follows:

(1) $$I_{eye}=D\cdot I_{source}.$$

That is, the human eye only perceives the signal’s average power. Consequently, it is possible to control this intensity by appropriately reducing the duty cycle of the AMOLS. In this paper, we explore the relationship between the effectiveness of a duty cycle during a direct camera attack and the ability to distinguish the AMOLS implementation. First, we review the effect of AMOLS on the camera and determine the irradiance needed to produce the desired disruptive effect on the camera. Next, we evaluate the dazzling irradiance on the human eye and determine the conditions that influence the eye’s ability to perceive and recognize the light source. Finally, after establishing the irradiance requirements, we examine the efficiency of image distortion caused by the designed pulse sequence on a well-known classifier, the Residual Neural Network (ResNet50) architecture, through simulations and experiments.

## 3. Materials and Methods

### 3.1. Dazzle Effect with Rolling Shutter Camera

**Figure 2:**
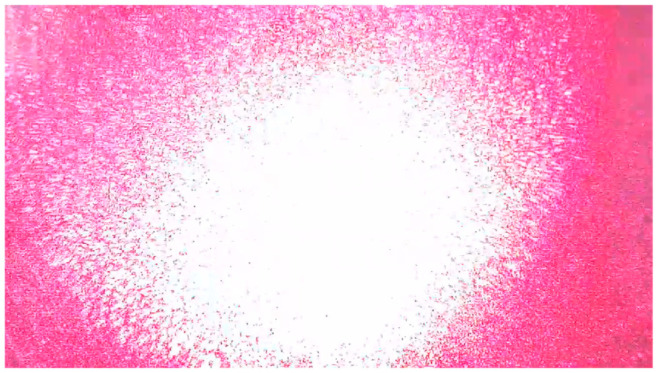
Experimental PSF measurement. The camera’s response to placed point source within the field of view. The radiant flux measured in the object plane is ~50 mW/cm^2^.

The spatial spread of a point source in the image plane is conventionally described by the diffraction of the Point Spared Function (PSF), generally given by the Fourier transformation of the entrance pupil. However, particularly for bright power sources (e.g., a laser source), other effects such as stray light scattering and halo [25] may occur in addition to the PSF diffraction, which may be considerably more significant than the PSF. The dazzling effect is demonstrated in Figure 2, where the measurement is acquired from a laptop camera (installed on a DELL-INSPIRON laptop with 0.92 Megapixel, 88° diagonal viewing angle). The AMOLS average power was 5 mW with ~3.5 mm spot diameter. As shown in Figure 2, a notable dazzling effect is observed when utilizing such a power level.

Previous studies on infrared imagers [26,27] have empirically shown that the diameter of the saturated area in the image plane, denoted as $x_{sat}$, can be approximated as follows:

(2) $$x_{sat}\propto {\left(\frac{I_{0}}{I_{sat}}\right)}^{\frac{1}{3}}$$

where $I_{0}$ and $I_{sat}$ are the laser irradiance and the saturation level, respectively. Based on results for visible light using a CMOS camera [28,29] a minimum average irradiance of $50\;mW/cm^{2}$ during each row exposure is required, and at least $0.1\;mW/cm^{2}$ pick irradiance to achieve dazzling with shorter pulses. We experimentally found that similar conditions hold for the camera used in this work, as observed in Figure 2.

**Figure 3:**
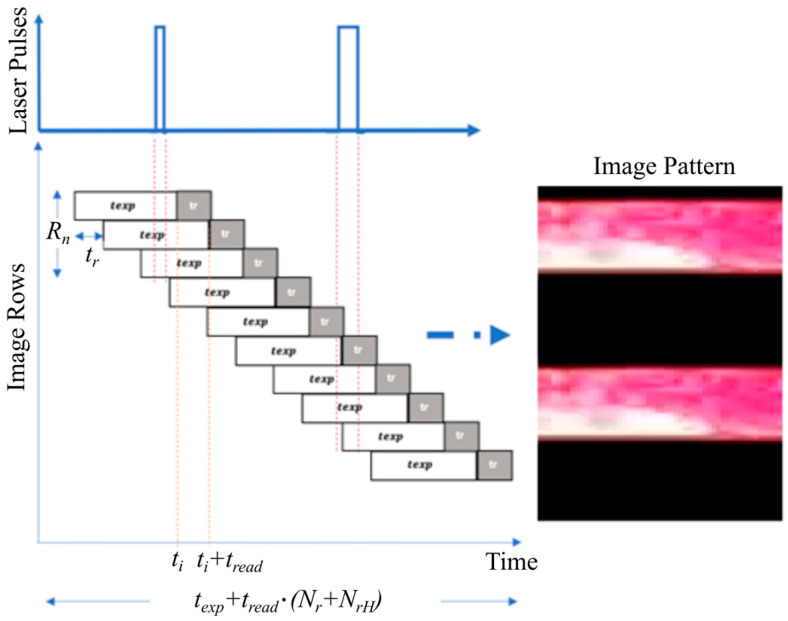
A schematic illustration of the rolling shutter effect caused by dazzling AMOLS. The rolling shutter mechanism transforms the temporal signal with a designed sequence of laser pulses (marked in blue at the top) into spatial distortion. This distortion occurs during different periods of reading and exposure for the pixel rows in the frame (indicated by white and gray blocks). As a result, a stripe-like pattern emerges in the acquired image (right).

Next, the dazzling effect formed in the attacked image is examined. With a rolling shutter camera, every row in the frame collects ambient light during different periods. As shown in Figure 3, the i-th row of the sensor records the light integrated during the period from $t_{i}-t_{exp}$ till $t_{i}$, while for the following row i+1, the integration time will be until $t_{i}+t_{read}$, where $t_{read}$ denotes the reading time of a single row and $t_{exp}$ denotes the exposure time of a single row. The duration of scanning each frame, denoted by $t_{frame}$, can be expressed as follows [16]:

(3) $${t_{frame}=t}_{read}\left(N_{r}+N_{rH}\right)+t_{exp}.$$

where $N_{r}$ and $N_{rH}$ are the number of pixel rows and the number of hidden pixel rows in each frame, respectively.

**Figure 4:**
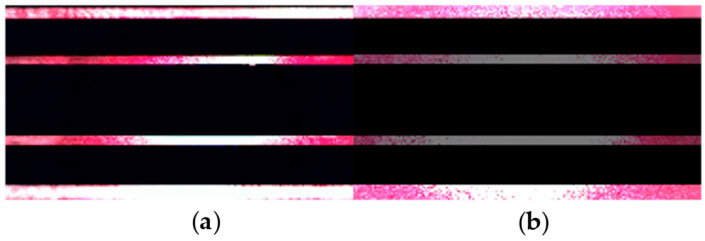
Dazzle effect of rolling shutter sensor by a modulated light source. (**a**,**b**) The resulting dazzle pattern for AMOLS via (**a**) experiment and (**b**) simulation with $R_{n}=37$.

The ratio $R_{n}=t_{exp}/t_{read}$ determines the number of exposed rows at any given time (see Figure 4). Thus, $R_{n}$ is referred to as the row’s exposure constant. It is worth highlighting that if the pulse duration generated by the AMOLS is shorter than $t_{read}$, exactly $R_{n}$ rows will be dazzled, regardless of the pulse width. For instance, both pulses with a duration 1μs and 2μs will produce the same pattern when using a typical camera with a reading time of $t_{read}\approx 30\;\mu s$. The experimentally obtained dazzle pattern for the rolling shutter sensor when the AMOLS is applied is shown in Figure 4, along with the simulated stripe-line pattern utilizing $R_{n}=37$ obtained with a calibration process. The simulation result corresponds well with the experimental measurement, with a structural similarity of 93%.

### 3.2. Photopic Conditions for Invisibility

**Figure 5:**
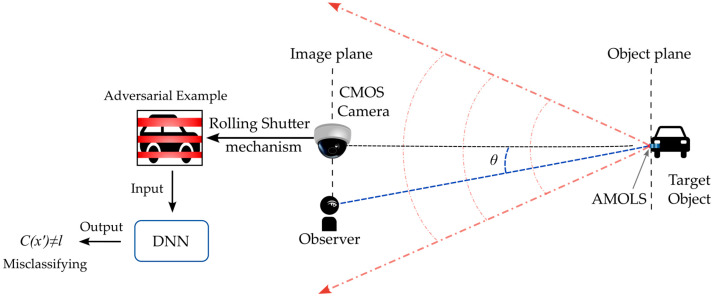
Invisible AMOLS implementation for direct camera attack. A target object (e.g., a car) is placed in the camera’s field of view, and a light source directly illuminates the camera (by sending a beam between the red arrows). The task of the DNN is to classify the acquired image. When applying the AMOLS, it must remain invisible to an observer at an angle θ relative to the optical axis.

This section focuses on determining the photometric conditions required to keep the AMOLS effectively invisible. The attack scenario is depicted in Figure 5, where a target object (car) and the AMOLS are placed in front of a camera while an observer is near the camera at an angle θ relative to the optical axis. The acquired image is then fed to DNN to classify the target. Consider that the AMOLS power is set to produce an irradiance of $e=50\;mW/cm^{2}$ at the sensor plane when active. The average power E received by the observer’s eyes from the AMOLS is influenced by the duty cycle of the AMOLS during the frame exposure period. The light source duty cycle denoted by D determines the average power of the light source, which can be expressed as E=e⋅D. In addition, assuming the AMOLS is smaller than the human eye’s angular resolution, the strictest condition would be the concentration of the seen power from any given source. Thus, a larger angular extent covered by the AMOLS would yield a lower peak power.

By denoting the background brightness by $L_{b}$ and the AMOLS brightness by $L_{AS}$, the contrast can be given by the following:

(4) $$C=\frac{L_{AS}-L_{b}}{L_{b}},$$

Previous studies by H.R. Blackwell [30] and W. Adrian [31] investigated the threshold contrast $C_{thr}$ required to detect an object. According to W. Adrian, a target contrast of 1 at a small angle is sufficient to recognize the target. Since radiance is a physical quantity conserved throughout an optical system, it dictates the brightness. When the solid angle covered by the target is smaller than the system’s resolving power, the AMOLS brightness has the following form [32]:

(5) $$L_{AS}=E\cdot 683\cdot V_{\lambda }\cdot \Omega_{eye}^{-2}\;\;\;\;\left[cd\cdot m^{-2}\right],$$

where $V_{\lambda }$ denotes the photopic efficacy and $\Omega_{eye}$ is the resolving power of the human eye (representing the strictest condition regarding the received power). Employing a camera model to represent the eye model, C.A. Williamson and L.N. McLin [33,34] proposed a scattering function based on empirical findings by J. Vos et al. [35], with an effective solid angle collected by the eye:

(6) $$f_{eye}\left(\theta ,A,p,L_{b}\right)=S\cdot L_{b}^{T}{\cdot g}_{eye}\left(\theta ,A,p\right)\;\left[sr^{-1}\right],$$

where $g_{eye}$ can be determined by the off-axis angle θ (see Figure 5), the age *A* (in years), and the eye pigment p, which is given by the following:

(7) $$g_{eye}\left(\theta ,A,p\right)=\frac{10}{\theta^{3}}+\left[\frac{5}{\theta^{2}\;}+\frac{0.1p}{\theta }\;\right]\left[1+{\left(\frac{A}{62.5}\right)}^{2}\right]+0.0025p\;[sr^{-1}],$$

Substituting the term of the average power E, and the angular resolution by $f_{eye}$, the AMOLS brightness expressed in Equation (5) takes the following form:

(8) $$L_{AS}=e\cdot D\cdot 683\cdot V_{\lambda }\cdot f_{eye}\;\left[cd\cdot m^{-2}\right],$$

Finally, by substituting Equation (8) into Equation (4), the light source duty cycle can be expressed by the following:

(9) $$D=L_{b}^{1-T}\frac{C_{thr}\left(L_{b}\right)+1}{e\cdot 683\cdot V_{\lambda }\cdot S\cdot g_{eye}\left(\theta ,A,p\right)}.$$

**Figure 6:**
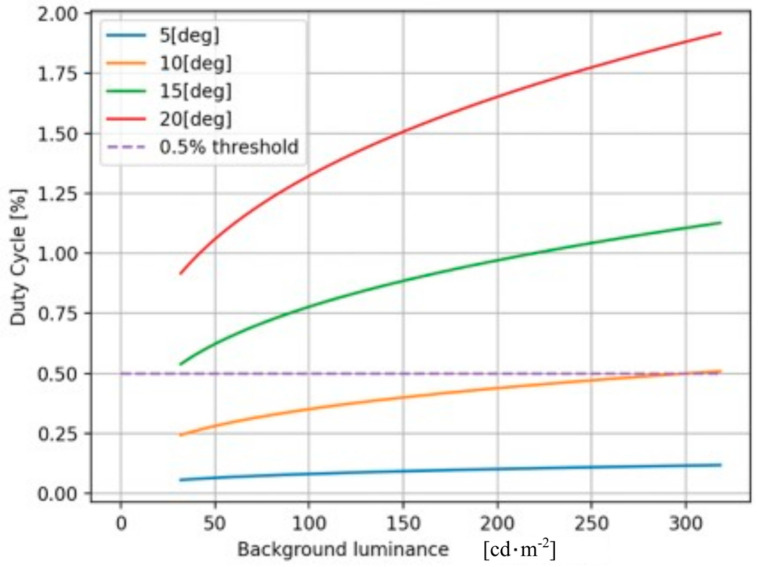
The duty cycle D of the AMOLS at the threshold of human discrimination as a function of the background luminance for various viewing angles θ.

Figure 6 shows the light source duty cycle D required for dazzling as a function of the background illumination for various viewing aspect angles. As the aspect angle increases, the effective radiance on the retina decreases. Consequently, the contrast decreases with the increasing background brightness, requiring more power to exceed the threshold. It is observed from the results shown in Figure 6 that for observers placed at an angle greater than 10 degrees, a duty cycle of 0.5% is sufficient to keep the source invisible, regardless of the background illumination level. The following sections will present a technique that can be operated even at lower duty cycle percentages.

### 3.3. Generating the Physical Adversarial Attack

Following the formalism in [2], the problem of finding an adversarial example can be formally defined as follows:

(10) $$\mathrm{minimize}\;{{\left|\left|x^{\prime }-x\right|\right|}_{2}}^{2}\;s.t.\;C\left(x^{\prime }\right)\neq l\;x^{\prime }\in {\left[0,1\right]}^{n},$$

where x is the undistributed image, $x^{\prime }$ is the perturbated image, l represents the ground truth label of the image x, and C(x) denotes the DNN used as a classifier. Periodically, solving such a problem can be incredibly complex, which leads to solving a more straightforward problem instead, as suggested in [36]. In brief, the goal is to find a small perturbation $\delta =x^{\prime }-x$, which can be applied to an image x to alter its classification while ensuring that the resulting image remains valid. Considering that the Softmax $V_{n}$ is applied on top of the DNN logits, the loss function mapping an image x to a positive real number can be described as follows

(11) $$f\left(x\right)=Loss_{C,\;l}\left(V_{n}(x)\right),$$

Accordingly, instead of formulating the constraint minimization problem as in Equation (10), one can use an alternative formulation and solve the following problem:

(12) $$\mathrm{minimize}\;{\left|\left|\delta \right|\right|}_{0}-\alpha \cdot f(x+\delta )\;s.t.\;x+\delta \in {\left[0,\;1\right]}^{n}.$$

where α represents the ratio between the magnitude of the disturbance and its effect’s intensity on the output, and $∥\cdot {∥}_{0}$ denotes the zero norm.

In our case, we aim to establish a relation between the pulsed laser activity and the resulting adversarial perturbation caused by the rolling shutter mechanism of the CMOS camera. This mechanism converts the temporal signal of the designed laser pulse sequence into a spatial distortion within the acquired image. $E_{eff}$ is an N-dimensional binary row vector representing the pulsed laser activity, which can be expressed by $N=\left(N_{r}+N_{rH}\right)/R_{n},$ where $R_{n}$ denotes the number of dazzled pixel rows by each pulse and $\left(N_{r}+N_{rH}\right)$ indicates the sensor’s total number of pixel rows (see Section 3.1). Specifically, a unit value at the i-th component of this vector $E_{eff}\left[i\right]=1,$ indicates a pulse occurring at the time $t=i\cdot t_{frame}/N$, and dazzles the sensor’s pixel rows from $i\cdot R_{n}$ to $(i+1)\cdot R_{n}.$ Thus, the indices of the dazzled pixel rows in the acquired image can be obtained by substituting each unit entry of the pulsed laser activity vector $E_{eff}^{T}$ with a size $R_{n}$ vector of ones, which is given by the following:

(13) $$E_{r}^{T}=E_{eff}^{T}⨂1_{R_{n}}^{T}$$

where ⨂ is the Kronecker product and $1_{R_{n}}^{T}$ is an $R_{n}$-dimensional column vector of ones. Consequently, $E_{r}^{T}$ is an $N\cdot R_{n}$-dimensional binary column vector in which unit entries indicate the dazzled sensor’s pixel rows. Next, the resulting dazzle pattern in the acquired N×M image (e.g., Figure 4) can be obtained by the following:

(14) $$\delta =E_{r}^{T}⨂1_{M}=\left(E_{eff}^{T}⨂1_{R_{n}}^{T}\right)⨂1_{M},$$

where $1_{M}$ is a size M vector of ones corresponding to the number of pixel columns in the acquired image. Instead of formulating the minimization problem following Equation (12), we now use an alternative formulation expressed in terms of the pulsed laser activity vector $E_{eff}$—the problem then becomes as follows: given x, find δ that satisfies the following:

(15) $$\mathrm{minimize}\;{\left|\left|E_{eff}^{T}\right|\right|}_{0}-\alpha \cdot f(x+\delta )\;s.t.\;\delta \in {\left[0,\;1\right]}^{n},$$

In practice, to implement a typical gradient-based optimization algorithm (such as SGD or ADAM) for solving Equation (15), we replace the binary vector derived from Equation (14). Rather than optimizing over the variable δ defined above, we change the variables and optimize over $\omega^{T}$, which has the following form:

(16) $$\delta =\left[\left(\frac{1}{2}\tanh\left(\omega^{T}\right)+1\right)⨂1_{R_{n}}^{T}\right]⨂1_{M},$$

where $\delta \in {\left[0,\;1\right]}^{n}$, and $\omega^{T}$ has the same dimensions as $E_{eff}^{T}$.

Since the exact moment of camera exposure is unknown to the attacker in a real-world setting, applying the AMOLS, consisting of a designed sequence of laser pulses, yields a dazzling pattern with a random horizontal shift. Considering the asynchrony between the attacking light pulse sequence and the camera’s exposure moment, we utilize the Expectation over Transformation (EoT) method [10] as follows:

(17) $$\mathrm{minimize}\;E_{t_{0}\sim T}\left\{{\left|\left|E_{eff}^{T}\right|\right|}_{0}-\alpha \cdot f\left(x+\delta \right)\right\}.$$

where T is the space of all possible instances of frame exposure, denoted as $t_{0}$.

## 4. Results and Discussion

This section presents the feasibility of conducting invisible adversarial attacks on DNNs in the physical domain by dazzling the camera. In addition, we evaluate the AMOLS performance using optimal dazzle patterns following the method described in Section 3.3, considering the pulsed laser activity depicted in Section 3.1. In the following sections, we employ both simulations and real experiments. First, we conduct simulations to investigate the effect of the AMOLS duty cycle while maintaining a constant pulse width. Next, we optically demonstrate the attack and examine its sensitivity to the pulse width.

### 4.1. Effectiveness of the AMOLS

**Figure 7:**
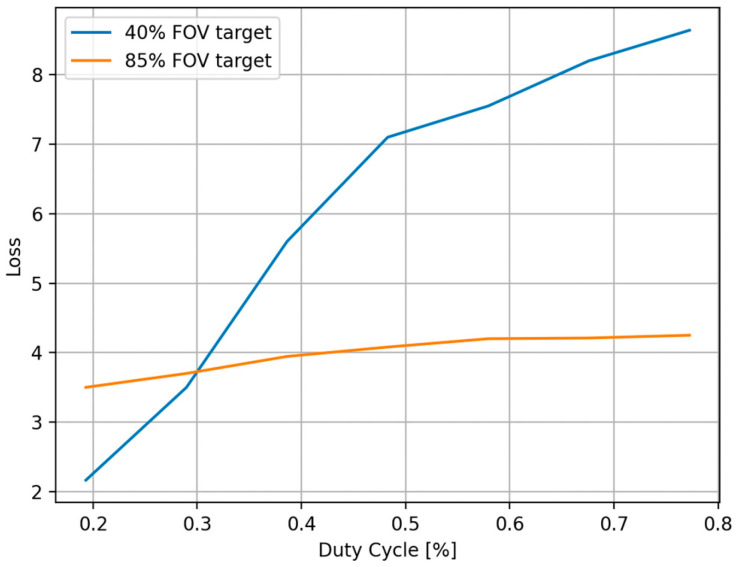
The effectiveness of the proposed attack on the loss function and its dependency on the duty cycle D of the pulsed laser beam.

We evaluate the effectiveness of the AMOLS based on the duty cycle of a pulsed laser (as discussed in Section 3.2) while keeping a constant pulse width. The ResNet50 classifier [37] and the standard cross-entropy loss function are utilized to simulate the adversarial attacks on the image classification model. Figure 7 shows simulation results of the loss function depending on the AMOLS duty cycle for two cases: where an object covers (1) approximately 40% of the field of view (FOV), and (2) approximately 85% of the FOV. These results focus on the “Coffee mug” as the target object, with the highest obtained values for each examined duty cycle as a result of optimizing the attack (as discussed in Section 3.3). It can be empirically determined that loss function values exceeding 2 exhibit poor classifier performances, resulting in misclassification across a significant number of input images—specifically, this enhances the effectiveness of the AMOLS. The results presented in Figure 7 indicate that when the duty cycle is set lower than 0.2%, the attack remains feasible—yet the classification model tends to yield better results when the target object covers ~40% of the FOV. Conversely, increasing the AMOLS duty cycle substantially raises the loss, thereby enhancing the effectiveness of the attack in the case of an object occupying ~40% of the FOV. Additionally, for a target object that covers ~85% of the FOV, the attack proves effective across the entire duty cycle range examined, with a milder dependence on changes in the AMOLS duty cycle.

**Figure 8:**
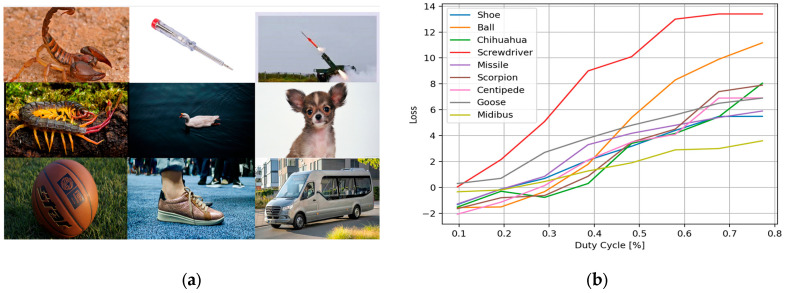
The AMOLS is applied to different objects. (**a**) Examples of attacked images. (**b**) The dependence of the loss function on the attacking light source duty cycle for various objects.

In addition, we examined a range of target objects during the attack, imaged from various angles of view corresponding to different classes—several samples are shown in Figure 8a. An analysis of the effect of the AMOLS duty cycle, while maintaining constant pulse width, on the classifier’s loss function across diverse input images is shown in Figure 8b. We empirically found the critical values of the cross-entropy loss function at which the DNN begins to misclassify objects across different classes, considering an offset in the obtained loss curves above these critical values. It is observed from the results shown in Figure 8b that an AMOLS duty cycle of 0.4%, which corresponds to a designed sequence of 4 laser pulses, successfully fools the classifier in all cases.

### 4.2. Real Experiments on Physical-World Adversarial Attack

We carried out real experiments to evaluate the feasibility of the proposed optical-based physical adversarial attacks in real-world scenarios formed by converting the light temporal signal to a spatial distortion within the acquired image. A coffee mug is used as the target object and placed inside the FOV of a laptop camera (installed on a DELL-INSPIRON laptop with 0.92 Megapixel, 88° diagonal viewing angle). For the attack, a pulsed laser beam is directed at the camera from a position adjacent to the object, produced from a 650 nm dot diode laser, with an average power of 5 mW and a spot size of 3.5 mm. A sequence of pulses is designed to generate the adversarial dazzle pattern following the optimization method described in Section 3.2, where the temporal modulating signal is produced utilizing the Arduino-Uno microcontroller board. The camera captures both the light reflected from the object and the light emitted by the AMOLS. The acquired images are then fed to the DNN for classification. We conducted our experiments with no ambient light, as this represents the most challenging condition for our problem setting, which requires the light source to remain invisible to a human observer. As illustrated in Figure 6, as the background illumination decreases, the allowable AMOLS illumination budget that can remain invisible also decreases. Conversely, a lower AMOLS illumination budget challenges the success of attacks, as indicated by the reduced classification loss shown in Figure 8b.

**Figure 9:**
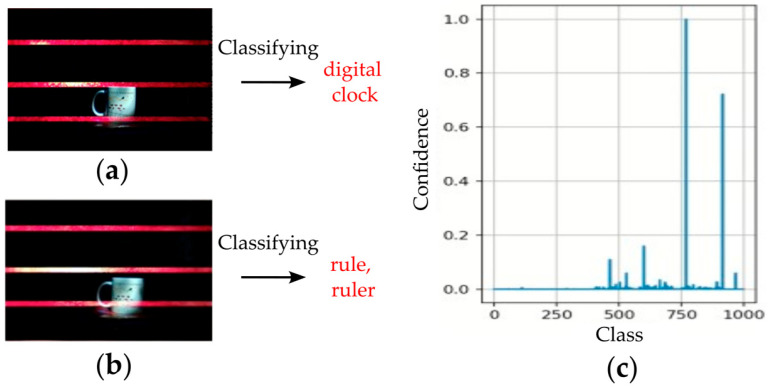
Results of AMOLS realization on an image classification model. Physical-world adversarial examples generated via two shots recording when setting different AMOLS activities: (**a**) pulse width of 1 μs with D=0.01%, and (**b**) pulse width of 70 μs with D=0.85%. (**c**) The DNN’s confidence in the predicted results across the 1000 classes it was trained on, with the index for the correct “coffee mug” label being #500.

Figure 9a,b shows two optical-based physical adversarial examples and their corresponding predictions from the image classification model. These examples were generated from two separate exposure shots, where the AMOLS used different pulse widths. It is worth mentioning that the attacking light pulse sequence is not synchronized with the camera’s exposure moment (see Section 3.3), leading to variations in the dazzle pattern across each frame, specifically introducing a horizontal shift. Videos showing the footage from the attacked camera sequence are provided in the Supplementary Materials. Additional examples can be found on the GitHub repository associated with this paper at https://github.com/ZviSteinOpt/RollingShutterAttack/tree/main (accessed on 1 April 2025). The invisible CMOS camera dazzling attack induces misclassification across the input images, significantly reducing the classifier’s confidence in the correct 500th class, as shown in Figure 9c.

**Figure 10:**
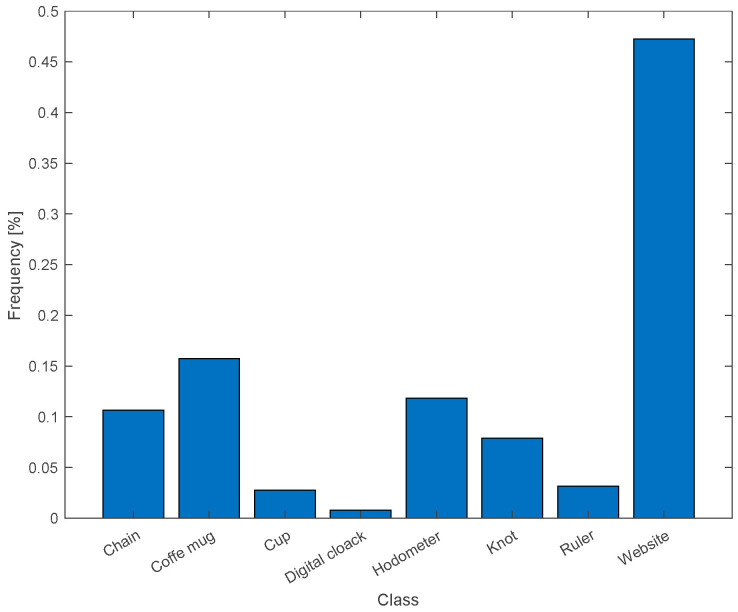
The frequency distribution of the DNN predictions during the attack. While the object’s correct label is a “coffee mug”, the attack exhibits an attack success rate of 85%.

**Figure 11:**
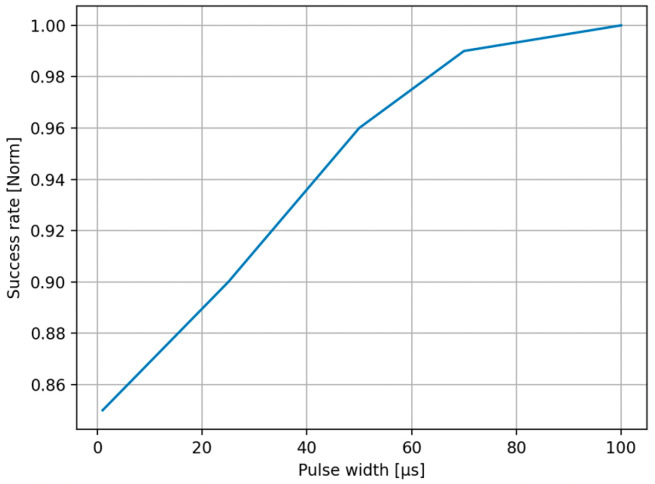
The average attack success rate as a function of the AMOLS pulse width.

The distribution of predictions made by a targeted DNN model across various classes during the optical-based physical adversarial attacks is depicted in Figure 10. It is based on 254 repeated trials, where the AMOLS operates four pulses, having a pulse duration of 1 μs. The results indicate that the designed attack achieved an 85% success rate under these conditions. The results shown in Figure 11 indicate that a higher attack success rate can be achieved by increasing the pulse width. When the AMOLS pulse width exceeds approximately 70 μs, the physical-world attack success rate approaches 98%. However, following Section 3.2, increasing the pulse width reduces the range of concealed viewing angles (see Figure 6). These exhibit a tradeoff between the angular realm achieving invisibility and the success rate of the physical-world attack as the AMOLS duty cycle varies. Considering that the camera captures 30 frames per second, a pulse of 1 μs corresponds to a low duty cycle of 0.012% ($D=100\cdot 4\cdot 1\;\mu s\cdot 30s^{-1}=0.012\%$), whereas a pulse duration of 70 μs results in a higher duty cycle of 0.84%. It can be observed from the results shown in Figure 6 that setting a duty cycle of 0.01% ensures the AMOLS activity remains invisible to the observer located at angles greater than approximately 5° from the optical axis. In comparison, a duty cycle of 0.85% could be sufficient to maintain the invisibility of optical-based physical adversarial attacks at a viewing angle of 15°.

The performance and properties of our attack are summarized in Table A1 in Appendix A, together with a comparison to that of other physical adversarial attacks involving image sensors.

## 5. Conclusions

In summary, we introduced a novel method for conducting optical-based physical adversarial attacks on DNN. The attack is demonstrated by directing a pulsed light at a CMOS camera. The rolling shutter mechanism of the camera converts the temporal signal, which consists of the designed sequence of light pulses, into a spatial distortion within the physical-world adversarial image. The photometric conditions and light pulse characteristics are analyzed to dazzle the CMOS camera sufficiently, thereby fooling the DNN model while keeping the AMOLS activity invisible to observers in the environment.

We demonstrated that the light source duty cycle enables the control of the tradeoff between the attack’s success rate and the required angular degree of concealment. For instance, with the proposed method, an 85% success rate for the physical-world attack can be achieved while ensuring the invisibility of light source activity to the observer except for a narrow angular range of 5° from the optical axis. However, the attack success rate could be increased to 98% by allowing a slight reduction of 10° in the angular concealment range.

## Data Availability

The original data presented in the study are openly available in the GitHub repository at https://github.com/ZviSteinOpt/RollingShutterAttack/tree/main (accessed on 1 April 2025).

## Supplementary Materials

The following supporting information can be downloaded at: https://www.mdpi.com/article/10.3390/s25072301/s1. The following supporting videos show the footage from the attacked camera sequence when setting different AMOLS activities. Video S1: pulse width of 1 μs with D=0.01%, and Video S2: pulse width of 70 μs with D=0.85%.

## Appendix A

**Table A1:** Comparison of physical adversarial attacks involving the image sensors.

Physical World Attack	Attack Mechanism	Targeting Camera Sensors	Adversary Physical Access	Achievable Attack Success Rate	Invisibility Criterion
EM Injection [12]	CCD interface	✓	X Near distances	50%~94% ^*a*^	✓
AdvLB [13]	Spatial laser beam	X	X	77.43%~100% ^*b*^	X
CamData Lane [14]	Camera data lane	X	✓ Camera interface	89.2%~96% ^*c*^	−
RS Backdoor Attack [18]	CMOS dazzling	✓	X	40%~88% ^*d*^	X ^*f*^
Adversarial RS [19]	CMOS dazzling	✓	X	~84%	X ^*f*^
Our Attack	Invisible AMOLS	✓	X	85%~98% ^*e*^	✓

^*a*^ Average performance from various viewpoints depending on the threat model. ^*b*^ Depending on indoor or outdoor attacks. ^*c*^ Depending on the DNN model. ^*d*^ Based on simulation study or physical-domain study. ^*e*^ Depending on the observer zone location restriction (Figure 5). ^*f*^ Designed to prevent visible flickering, although the illumination source may be seen shining. RS—Rolling Shutter. X and ✓ represent whether the attacks target the camera sensors to inject their perturbations, require physical access by the adversary, or satisfy the invisibility criterion in the physical domain. — represents a designed attack assuming adversary physical access.
